# The complete chloroplast genome of *Polygonatum odoratum* (Liliaceae), an endemic medicinal herb

**DOI:** 10.1080/23802359.2020.1834888

**Published:** 2020-11-11

**Authors:** Ze-Huan Wang, Ya-Qiong Li

**Affiliations:** Faculty of Traditional Chinese Pharmacy, Yunnan University of Traditional Chinese Medicine, Kunming, Yunnan, People’s Republic of China

**Keywords:** Complete chloroplast genome；*Polygonatum odoratum*；phylogenetic analysis；Liliaceae

## Abstract

In this study, we sequenced the complete chloroplast genome of *Polygonatum odoratum* with Illumina sequencing technology. The complete chloroplast genome length is 156,082 bp shows a typical tetrad structure, which manifests as one large and one small single copy (LSC and SSC) regions of 85,009 and 18,513 bp, isolated by two inverted repeat regions (IRs) of 26,280 bp. This study annotated altogether 131 unique genes, consisting of 86 protein-encoding genes, 8 rRNA, and 38 tRNA. According to the maximum likelihood phylogenetic tree based on eight complete chloroplast genomes, *P. odoratum* shows a close association with additional *Maianthemum* genus. The chloroplast genome-wide for *P. odoratum* would help to conserve the precious natural populations.

*Polygonatum odoratum* (Mill.) Druce, a typical representative of the Liliaceae family, is a perennial herbaceous plant that is widely distributed in East Asia and Europe (Zhao et al. 2018). *Polygonatum odoratum* has been found to contain several components with bioactive effects, including polysaccharides, steroidal glycosides, dipeptides, flavonoids, amino acids, and trace mineral elements (Lin et al. 1994; Deng et al. 2012). *Polygonatum odoratum* rhizomes are regarded as the medicinal parts of the plant, and have been used extensively to treat diseases such as rheumatic heart disease, hypoimmunity, cardiovascular diseases, and diabetes (China Pharmacopoeia Committee 2015). *Polygonatum odoratum* has been recognized as the endemic medicinal perennial herb, because resources of this herb are diminishing due to uncontrolled harvesting. Therefore, it is necessary for us to learn more about its genetic data and pay more attention to it. Notably, the chloroplast genome-wide for *P. odoratum* would help to conserve the precious natural populations.

In this study, silica-gel-dried leaves of *P. odoratum* were collected from Xianggelila of Yunnan Province, China (99°39.844E, 27°53.684N), and voucher specimens (PO201909001) were deposited in the Herbarium of Yunnan University of Chinese Medicine. Genomic DNA was extracted with the CTAB method (Doyle and Doyle 1987). For Illumina sequencing, at least 2 μg genomic DNA was used for each sample in sequencing library construction. Paired-end libraries with the insert size of 400 bp were constructed according to the manufacturer’s instructions (Biooscientific, AIR™ Paired-End DNA Sequencing Kit, Austin, TX) and then sequenced on Illumina Hiseq X-Ten (Illumina, San Diego, CA). There are 2.16 GB of sequence data was generated. The reads of the complete chloroplast genome were assembled using *de novo* assembling constructed in SPAdes version 3.9.1 (Bankevich et al. 2012), using kmer lengths of 21–105 bp; followed by reference guided assembling conducted with Bandage version 0.8.1 (Wick et al. 2015) and Geneious version 9.1.4 (Kearse et al. 2012). *Polygonatum humile* (GenBank: MN218691) was used as reference for annotation using GeSeq (Tillich et al. 2017), coupled with manual correction for boundaries.

The complete chloroplast genome of *P. odoratum* was 156,082 bp in length (GenBank accession number: MT646047), the GC content was 37.7%. Large single-copy (LSC) and small single-copy (SSC) regions contained 85,009 and 18,513 bp, respectively, while inverted repeat regions (IR) was 26,280 bp in length. A total of 131 unique genes were annotated, including 38tRNA, 8rRNA, and 85 protein-coding genes. Seven protein-coding genes, nine tRNA, and four rRNA genes were duplicated in the IR regions. In total, 18 intron-containing genes were in the chloroplast genome of *P. odoratum* of which three genes (*rps12*, *clpP*, and *ycf3*) include two introns and the rest include a single intron.

Six complete chloroplast genome sequences of *Polygonatum* and one complete chloroplast genome sequences of *Maianthemun* were downloaded from NCBI to further investigate the phylogenetic position of *P. odoratum*. We constructed a maximum likelihood tree using RAxML (Stamatakis 2014) after the sequences were aligned using MAFFT (Katoh and Standley 2013). The best nucleotide substitution model for constructing the phylogenetic tree is the Tamura–Nei model. Phylogenetic trees show that the species of *Polygonatum* were clustered into one branch and formed a monophyletic clade with 100% bootstrap support value (Figure 1). The cladistic clustering of *P. odoratum* and *P. odoratum* was close and had high support rate. The complete chloroplast genome of *P. odoratum* would help in understanding the genetic information and conserving the precious natural populations.

**Figure 1.**
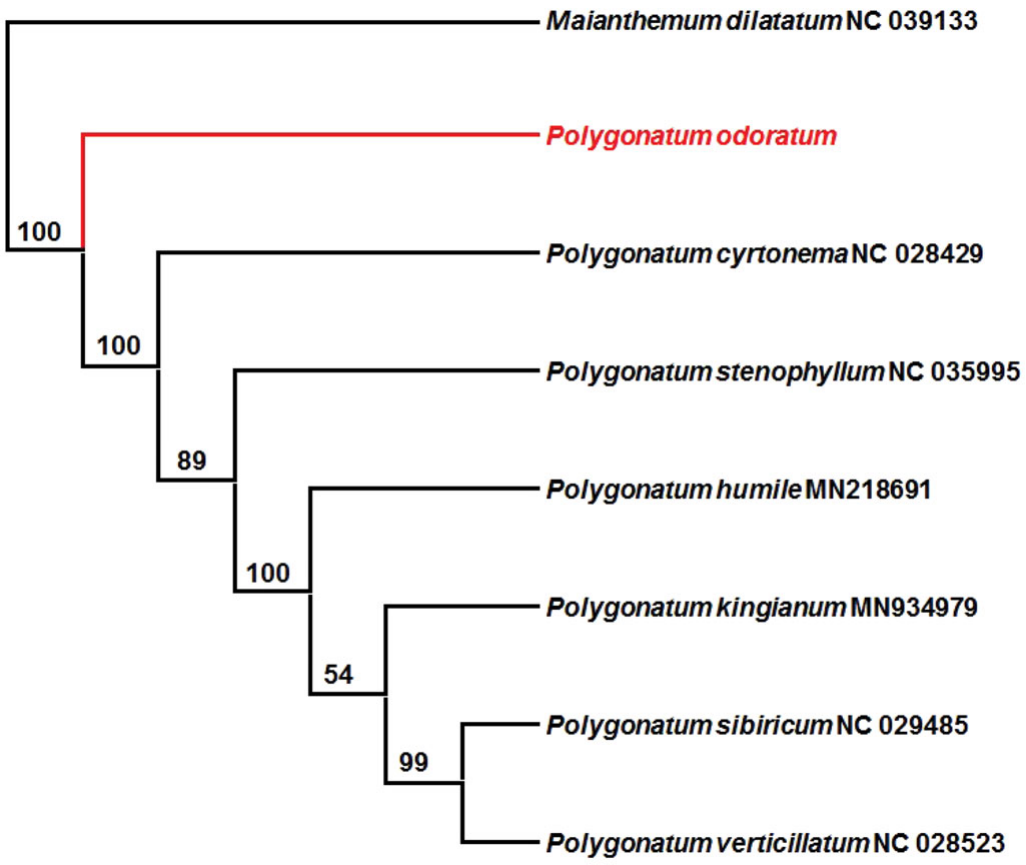
Maximum likelihood phylogenetic tree based on eight complete chloroplast genomes (bootstrap repeat is 1000).

## Data Availability

The data that support the findings of this study are openly available in GenBank of NCBI at https://www.ncbi.nlm.nih.gov, reference number MT646047.
